# The Association of Body Mass Index With the Risk of Pulmonary Hypertension in Adults: A Systematic Review and Meta-Analysis of Observational Studies

**DOI:** 10.3389/fmed.2021.680223

**Published:** 2022-01-25

**Authors:** Shoufang Pu, Lidan Yin, Bi Wen, Juan He

**Affiliations:** Department of Cardiology, West China Hospital, Sichuan University/West China School of Nursing, Sichuan University, Chengdu, China

**Keywords:** obesity, body mass index, pulmonary hypertension, systematic review, meta-analysis

## Abstract

**Backgrounds:**

Findings regarding the association of body mass index (BMI) with pulmonary hypertension (PH) are conflicting, and there is no systematic review and meta-analysis to summarize the results. Therefore, the purpose of this systematic review and meta-analysis is to assess this relationship.

**Methods:**

To detect the relevant articles, PubMed, Scopus, and Google Scholar were searched until February 2021. Included essays were pooled using a random-effect model. Cochrane *Q*-test and I^2^-test was applied to assess between-study heterogeneity.

**Results:**

Fourteen articles (eight cross-sectional and four cohort studies) were included in the meta-analysis. The meta-analysis of comparing highest vs. lowest BMI categories did not indicate a significant association between BMI and PH (Summary Effect Estimate: 1.59 (95% CI: 0.50, 5.07, I^2^ = 92.3). Furthermore, The summary risk estimate for a one-unit increment in BMI was 1.01 (95 % CI: 0.99, 1.03), with high heterogeneity, I^2^ = 73.5 %, P heterogeneity <0.001). Subgroup analysis showed significant positive association between BMI and the risk of PH in studies controlled for cofounders, and studies with higher sample sizes (≥2,000).

**Conclusion:**

There is no significant association between BMI and risk of pulmonary hypertension. Further studies are required to confirm these findings.

## Introduction

Pulmonary hypertension (PH) is characterized by pulmonary vascular remodeling, which leads to an elevation in pulmonary vascular resistance (PVR), potentially eventuating in right heart failure and mortality (1, 2). The prevalence of pulmonary arterial hypertension (PAH) has been estimated from 11 to 26 cases per million adults (3). Some risk factors, including using drugs and toxins such as amphetamines, diseases such as HIV infection, portal hypertension, pregnancy, and obesity were attributed to PAH development (4, 5).

The prevalence of overweight and obesity has considerably increased in recent years (6). The body mass index (BMI) is an indicator of increased body fat, and WHO considered it a good tool to measure obesity (7). Epidemiological data propose that elevated BMI affects the development of PH (8), and almost two-thirds of patients with PH are overweight or obese at the time of diagnosis (9). The data suggested that the mechanism of PH in obese persons includes endothelial dysfunction, obstructive sleep apnea, expansion of fatty tissue surrounding the pulmonary artery, anorexigen use, obesity hypoventilation syndrome, pulmonary thromboembolic disease, cardiomyopathy of obesity, and hyperuricemia (10).

Animal studies showed that metabolic disease due to obesity might lead to pulmonary vascular remodeling and precapillary PH (11). Epidemiologic studies demonstrated conflicting findings regarding the association of BMI and obesity with the risk of PH. Some of them showed positive association (12–14), and others did not find any relationships (15, 16).

Given that PH is one of the causes of mortality and morbidity worldwide and the growing prevalence of obesity, clarifying the association between body mass index (BMI) would be of critical importance in providing more particular guidelines for prevention of PH. Based on our knowledge, there is no systematic review and meta-analysis to summarize the data regarding the association of BMI with PH. Therefore, this systematic review and meta-analysis has been formed to assess the association of BMI with PH in adults.

## Materials and Methods

The framework of the present study was designed following the guidelines of the Preferred Reporting Items for Systematic Reviews and Meta-Analyses (PRISMA) statement (17).

### Search Strategy

PubMed, Scopus, and Google Scholar were explored using relevant keywords in order to discover the related articles published up to February 2021. The search term was obtained from Medical subject headings (MESH) and related keywords as follows: [(Obesity OR “body mass index” [Mesh] OR Obesity/complications [Mesh] OR overweight [Mesh] OR adiposity [Mesh] OR “body mass index” [Title/Abstract] OR BMI [Title/Abstract] OR “fatness” [Title/Abstract] OR “Obesity/complications” [Title/Abstract] OR overweight [Title/Abstract] OR adiposity [Title/Abstract]) AND (“Hypertension, Pulmonary” [Mesh] OR “Pulmonary Hypertension” [Title/Abstract])]. No filters were applied when searching the databases. To prevent missing any articles, the reference lists of the included articles and relevant reviews were manually inspected.

### Inclusion Criteria

Two investigators assessed the titles, abstracts, and, when necessary, the full texts of the articles using the following inclusion criteria: (1) observational studies (cohort, cross-sectional, and case-control studies); (2) studies that reported the association of BMI with the risk of PH; (3) studies conducted on adults; (4) studies that reported the hazard ratio (HR), rate ratio (RR), or odds ratio (OR) and the corresponding 95% confidence interval (CI) in a linear or categorical manner; (5) studies published in the English language. If the two investigators encountered any paradoxes, they reached a solution through a discussion with the principal investigator.

Studies that did not report HRs, RRs, or ORs with the corresponding 95% CI, those used International Classification of Diseases (ICD) for obesity definition, studies with insufficient data, those conducted on children, and studies with similar populations were excluded. In the case the authors could not access the full text of a paper, they sought it by contacting the corresponding author. However, no responses were received.

### Data Extraction

Two independent authors read each paper carefully to extract the following information: first author's name, publication year, country, study design, the participants' age (mean/range), gender, health condition, total sample size, number of PH patients, the cut-off points for defining PH, PH assessment approach, duration of follow-up for cohort studies, continuous or categorical values, and the adjustments made. When two authors could not reach an agreement on an issue, the corresponding author resolved it.

### Quality and Risk of Bias Assessment

We evaluated the quality of each eligible study using the Newcastle-Ottawa Scale (NOS) (18). This scale comprises three parts, including selection, comparability, and exposure or outcome. The total score ranged between 0 and 9. In our study, papers with a score of seven or above were presumed as good quality. Risk of bias assessment was conducted using the Risk Of Bias In Non-randomized Studies of Exposures (ROBINS-E) tool (19). The ROBINS-E tool comprises seven domains through which bias might be introduced. The questions of these domains include (1) bias due to confounding, (2) bias in the selection of participants into the study, (3) bias in the classification of exposures, (4) bias due to departure from intended exposures, (5) bias due to missing data, (6) bias in the measurement of outcomes, and (7) bias in the selection of reported results. Studies were categorized as having a low, moderate, serious, and critical risk of bias under each domain. Two authors assessed the quality and risk of bias of the studies separately; if there was a discrepancy, they made a final decision after a discussion with the principal investigator.

### Statistical Analysis

The random-effects model was used to calculate estimated risk with 95% CIs to compare the highest vs. lowest BMI categories or combine the findings of the association of one-increment in BMI and PH risk. Cochrane *Q*-test and I^2^ test was applied to assess heterogeneity among included studies. Cochrane *Q*-test, with P < 0.1 expressing significant between-study heterogeneity. The I^2^ values of 25-50, 50-75, and >75% were considered as low, moderate, and high heterogeneity, respectively (20). Subgroup analysis was carried out based on the following variables: PH assessment tool, adjustment, country, sample size, number of cases, and quality of assessment. Inspection of the funnel plots for asymmetry and Egger test (*P* < 0.10) were implemented to identify publication bias (21). Sensitivity analysis was performed to assess the effect of each study on summary effect size. All statistical analyses were performed using STATA software version 15.1 (Stata Corporation, College Station, Texas, USA). The P-value below 0.05 was assumed as significant.

## Results

**Figure 1:**
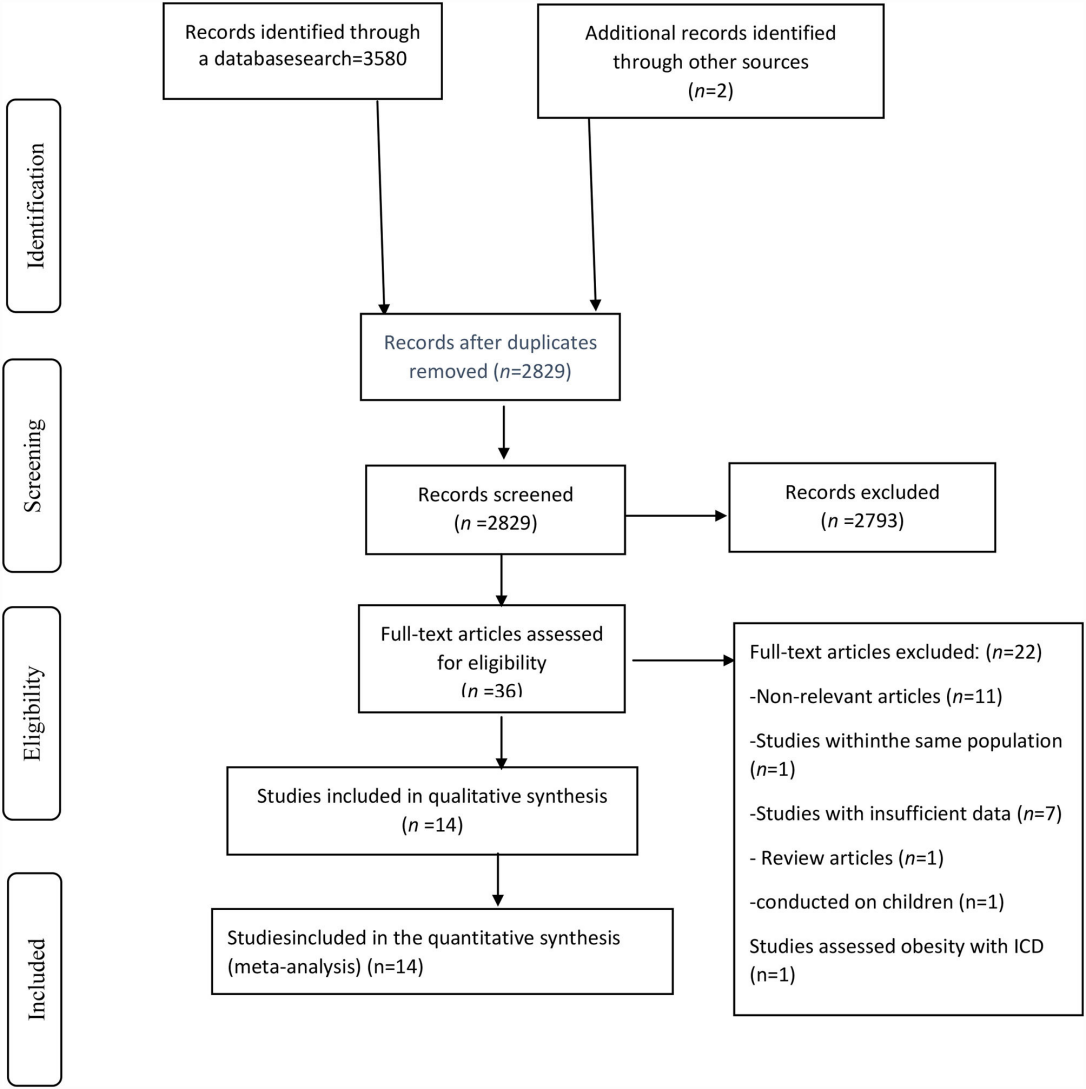
Study selection flow diagram.

A systematic search found a total of 3,582 records that 2,829 remained after removing duplicates (Figure 1). After evaluating essays based on the title and abstract, 2,793 publications were excluded. Among 36 remained publications, 22 papers were excluded for the following reasons: not relevant articles (*n* = 11), insufficient data (*n* = 7), review article (*n* = 1). Furthermore, studies that defined obesity with ICD instead of BMI (*n* = 1), those conducted on children (*n* = 1), and studies with similar population (*n* = 1) were also removed. Finally, six cohorts (16, 22–26) and eight cross-sectional studies (7, 12–15, 27–29) were chosen for the systematic review and meta-analysis.

### Study Characteristics

The demographic information of the eligible studies is presented in Table 1. A total of 22,173 participants and 7,689 PH patients ranging between 18 and 84 years were included in this review. Most of the relevant studies were performed in the United States (*n* = 8) (12–16, 24, 25, 29), while others were done in China (*n* = 3) (26–28), Portugal (*n* = 1) (22), Taiwan (*n* = 1) (23), and Iran (*n* = 1) (7). The studies were published between 2004 and 2020. PH was assessed by right heart catheterization (RHC) (*n* = 6) (13, 14, 16, 24, 25, 28) or echocardiography (*n* = 8) (7, 12, 15, 22, 23, 26, 27, 29). Two of the included papers had high methodological quality (score ≥ 7) (13, 27), while the others had low quality (<7) (Table 1). Based on the ROBINS-E tool, three publications had a moderate risk of bias and others had a serious risk of bias.

**Table 1:** Characteristics of observational studies eligible in the systematic review and meta-analysis.

**Code**	**References**	**Country**	**participants**	**Study design**	**Sample Size/case (gender)**	**Age**	**Exposure**	**PH definition**	**PH assessment method**	**Adjustments**	**NOS score**
1	Al-Naamani et al. (16)	US	patients with advanced lung disease	Cohort	399/137 (male and female)	≥18yr	BMI (continuous)	mPAP > 20mmHg PVR ≥ 3 wood units	RHC	age, sex, race/ethnicity, primary lung diagnosis, forced vital capacity (FVC), and PAWP	4
2	Luo et al. (28)	China	patients with suspected PH	Cross sectional	559/488 (male and female)	≥18yr	BMI (continuous)	mPAP ≥ 25 mmHg	RHC	No	4
3	Frank et al. (13)	US	patients undergoing right-sided heart catherization	Cross sectional	8,940/5453	18-80	BMI (continuous)	mPAP > 20 mmHg	RHC	age, sex, heart rate, hypertension, diabetes mellitus, obstructive sleep apnea, chronic kidney disease, previous myocardial infarction, and heart failure	9
4	Gou et al. (27)	China	highlanders	Cross sectional	1,129/70	≥18yr	BMI <24 kg/m^2^ BMI 24-28 kg/m^2^ BMI > 28 kg/m^2^	mPAP > 30 mmHg	Echocardiography	NR	8
5	Zhang et al. (26)	China	CKD patients	Cohort	705/331	≥18yr	BMI (continuous)	SPAP > 35 mmHg	Echocardiography	NR	2
6	Fekri et al. (7)	Iran	patients with COPD	Cross sectional	1,078/136	70.1 ± 12.2	BMI <18.5 kg/m^2^ BMI 18.5-24.99 kg/m^2^ BMI ≥ 25 kg/m^2^	mPAP ≥ 40mmHg	Echocardiography	No	5
7	Hsieh et al. (23)	Taiwan	patients on chronic hemodialysis and with heart failure	Cohort	160/51	68.8 ± 11.1	BMI (continuous)	SPAP > 35 mmHg	Echocardiography	diabetes, CAD, smoking, and ejection fraction	2
8	Barros et al. (22)	Portugal	intermediate-to-high risk PE	Cohort	213/15	61.1 ± 18.1	BMI (continuous)	SPAP > 40 mmHg	Echocardiography	NR	5
9	Choudhary et al. (12)	US	non-institutionalized adult American Africa	Cross sectional	3,282/223	35-84	BMI <25 kg/m^2^ BMI 25-30 kg/m^2^ BMI ≥ 30 kg/m^2^	trans-tricuspid gradient > 35 mmHg	Echocardiography	NR	6
10	Agarwal et al. (15)	US	hemodialysis patients	Cross sectional	288/110	≥18yr	BMI (continuous)	SPAP > 35 mmHg	Echocardiography	No	6
11	Leung et al. (14)	US	Elevated Pulmonary Venous Pressure and Preserved Ejection Fraction	Cross sectional	455/239	67.8 ± 11.2	BMI ≥ 40 kg/m^2^	mPAP ≥ 25 mmHg	RLHC	NR	5
12	Robbins et al. (24)	US	consecutive patients	Cohort	122/17	55.7 ± 12.1	BMI ≥ 30 kg/m^2^	mPAP≥25mmHg	RHC	No	3
13	Valencia-Flores et al. (29)	US	obese patients	Cross sectional	57/55	42.77 ± 12.1	BMI (continuous)	SPAP > 30 mmHg	Echocardiography	NR	4
14	Assad et al. (25)	US	patients with PH	Cohort	4,786/364	56 ± 14	BMI (continuous)	PAWP > 15 mmHg, DPG ≥ 7 mmHg	RHC	Age, sex	7

*BMI, Body mass index; CAD, chronic artery disease; CKD, Chronic Kidney patients; COPD, Chronic obstructive pulmonary disease; DPG, diastolic pressure gradient; ICD-9, International Classification of Diseases-9; mPAP, mean pulmonary arterial pressure; NR, not reported; NOS, Newcastle-Ottawa Scale; PAWP, pulmonary arterial wedge pressure; PH, pulmonary hypertension; PVR, pulmonary vascular resistance; SPAP, systolic pulmonary arterial pressure; RHC, right heart catheterization; RLHC, right left heart catheterization; US, united states*.

### Meta-Analysis

**Figure 2:**
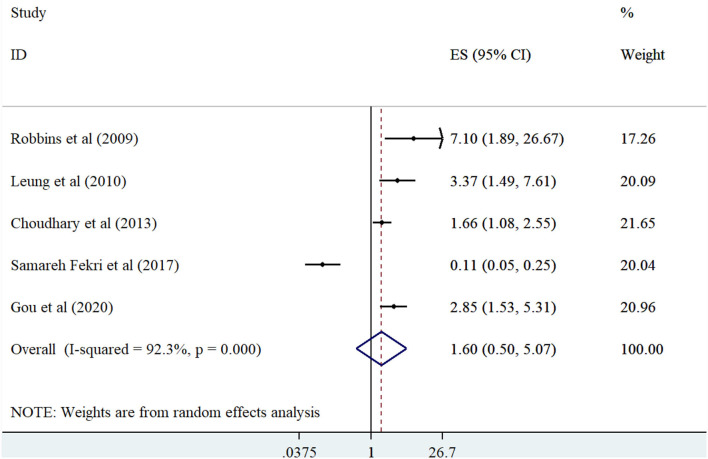
Forest plot derived from random-effects meta-analysis of studies investigating the association between BMI (high vs low) and pulmonary hypertension in adults. CI, confidence interval; ES, effect size.

**Figure 3:**
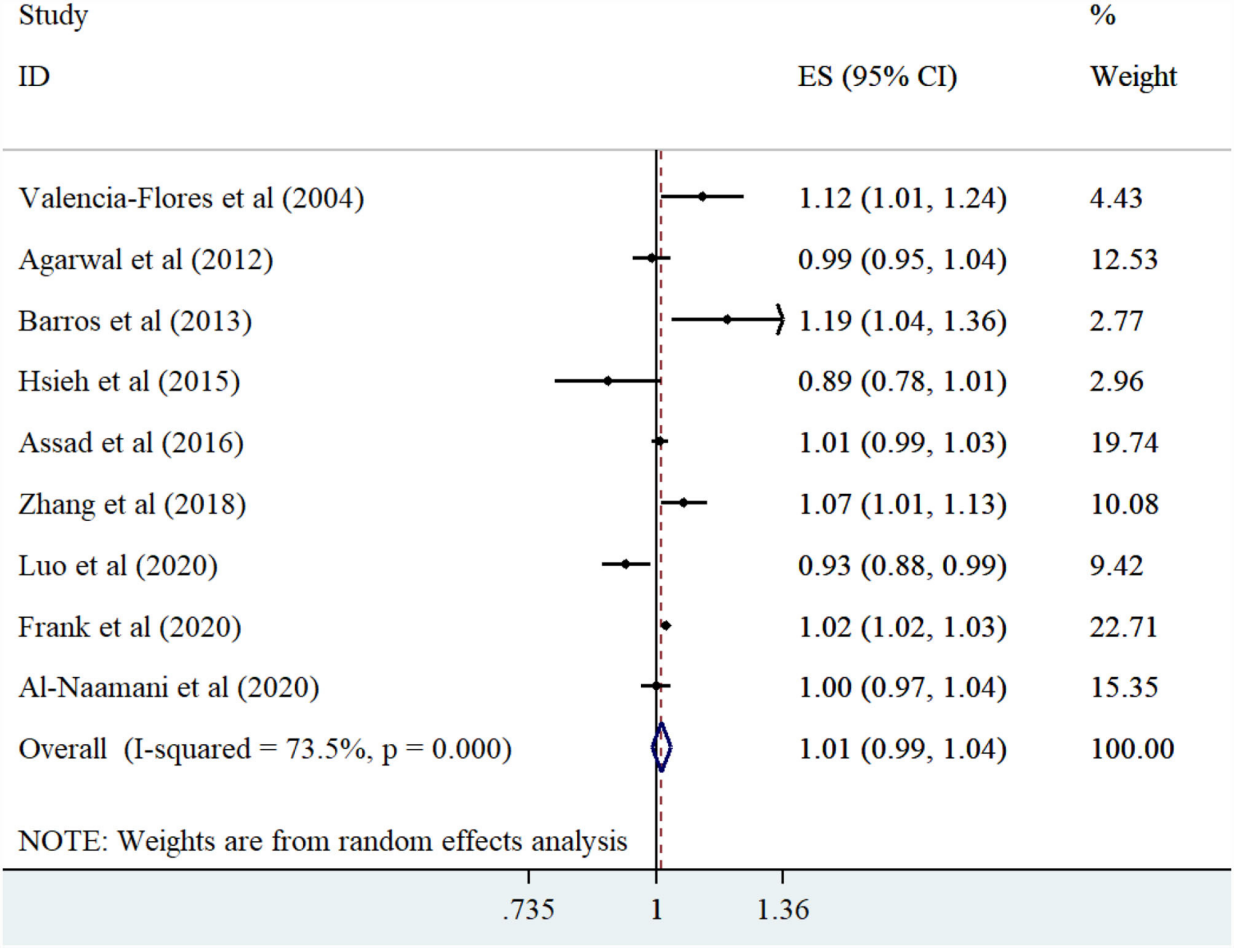
Forest plot derived from random-effects meta-analysis of studies investigating the association between one-unit increment in BMI with pulmonary hypertension in adults. CI, confidence interval; ES, effect size.

The meta-analysis of the five observational studies (7, 12, 14, 24, 27) indicated that people with highest BMI had not an increased risk of PH compared with people with lowest BMI (summary effect estimate: 1.59 (95% CI: 0.50-5.07, *I*^2^ = 92.3) (Figure 2). Furthermore, linear dose-response analysis of nine observational studies (13, 15, 16, 22, 23, 25, 26, 28, 29) demonstrated no significant association between one-unit increment in BMI and risk of PH (summary effect estimate: 1.01 (95% CI: 0.99, 1.03, *I*^2^ = 73.5%) Figure 3).

### Subgroup Analysis

In the analysis of comparing highest vs. lowest BMI categories, subgroup analysis showed PH assessment tool and adjustment were the source of heterogeneity. Furthermore, a significant positive association was observed between BMI and the risk of PH in the studies that assessed PH with RHC, were conducted in the United States, included adjustments, had larger sample sizes (≥2,000), and had a greater number of cases (≥200) (Table 2).

**Table 2:** Results of subgroup analysis for Body mass index and risk of pulmonary hypertension in adults.

**Group**	**Studies (*n*)**	**ES (95% CI)**	***P*-value**	***P*-within subgroups heterogeneity**	***I^**2**^* %**
**The highest vs. lowest comparison**					
Total	5	1.59 (0.50, 5.07)	0.426	<0.001	92.3
**PH assessment tool**					
RHC	2	4.13 (2.06, 8.27)	<0.001	0.347	0
Echocardiography	3	0.83 (0.17, 4.12)	0.82	<0.001	92.5
**Adjustment**					
Yes	3	2.30 (1.47, 3.59)	<0.001	0.187	40.3
No	2	0.85 (0.01, 50.72)	0.94	<0.001	96.4
**Country**					
US	3	2.85 (1.31, 6.20)	0.008	0.058	64.9
Non US	2	0.56 (0.02, 13.75)	0.724	<0.001	97.4
**Number of cases**					
<200	3	1.26 (0.11, 14.47)	0.848	<0.001	95.7
≥200	2	2.16 (1.10, 4.24)	0.024	0.132	56
**Sample size**					
<2,000	4	1.61 (0.28, 9.29)	0.593	<0.001	94.2
≥2,000	1	1.66 (1.08, 2.55)	0.021	-	-
**Linear dose-response association**					
Total	9	1.01 (0.99, 1.03)	0.305	<0.001	73.5
**PH assessment tool**					
RHC	4	1.01 (0.98, 1.03)	0.751	0.002	77.5
Echocardiography	5	1.04 (0.97, 1.12)	0.252	0.004	76
**Adjustment**					
Yes	7	1.02 (1.00, 1.05)	0.039	0.006	67.1
No	2	0.96 (0.91, 1.02)	0.219	0.101	62.8
**Country**					
US	5	1.01 (0.99, 1.03)	0.074	0.074	53.2
Non US	4	1.01 (0.90, 1.12)	0.853	<0.001	85.6
**Number of cases**					
<200	5	1.02 (0.96, 1.09)	0.456	0.006	72.1
≥200	4	1.01 (0.98, 1.04)	0.359	0.003	78.8
**Sample size**					
<2,000	7	1.01 (0.96, 1.07)	0.56	<0.001	76.6
≥2,000	2	1.02 (1.01, 1.03)	0.001	0.181	44

*AP, arterial pressure; BMI, Body Mass Index; ES, Effect Size; PH, pulmonary hypertension*.

Only the sample size was identified as the source of heterogeneity through subgroup analysis of the relationship between a one-unit increment in BMI and PH risk. Besides, subgroup analysis presented that a one-unit increment in BMI could elevate the risk of PH in adjusted studies, and studies with higher sample sizes (≥2,000) (Table 2).

### Sensitivity Analysis

Sensitivity analysis demonstrated that in either categorical or continuous analysis, no study significantly affected the overall effect size.

### Publication Bias

No evidence of publication bias was identified through both Egger's test and observation of the funnel plot regarding the association of a one-unit increment in BMI (*P* = 0.565) or highest vs. lowest BMI (*P* = 0.989) with the risk of PH.

## Discussion

In this study, we examined the association of BMI with PH in adults using eight cross-sectional studies and six cohort studies. This study failed to show any significant association between a BMI and risk of PH in adults.

Pulmonary hypertension is identified by elevated PAPs as well as increased pulmonary vascular resistance resulting in right ventricular failure (30). This study did not find any connection between BMI and the risk of PH in adults. In contrast to our findings, the results of the echocardiogram in a retrospective study indicated that 5% of healthy obese subjects (BMI > 30 kg/m^2^) had pulmonary artery systolic pressures of more than 50 mmHg (31). Taraseviciute et al. (32) demonstrated that 38% of postmenopausal women with PH and 48% of severe secondary PH postmenopausal women were obese (31). A cohort study with 4,176 young adults showed that BMI is associated with elevated PAP as assessed by echocardiography (33). Another cross-sectional study on 177 German patients with obesity hypoventilation syndrome found a positive association between BMI and mean PAP (34). A systematic review of nine interventional studies found that a median weight loss of 43 kg (range: 10-58 kg) could lead to a decrease in mean PAP over a median duration of 9.7 months (range: 0.75-23.0 months) (35). However, in another study on 93 patients with left ventricular systolic dysfunction, a low BMI appeared to be significantly related to PH and was considered an independent predictor of major adverse cardiac events (36). In another study on 26 idiopathic pulmonary artery hypertension (IPAH) patients, a BMI of <25 kg/m^2^ was the main predictor of high pulmonary artery pulse wave velocity (PA-PWV) (37). Varying assessment tools, adjustments, sample sizes, and numbers of cases may account for the varying results regarding PH.

Right heart catheterization (RHC) is the gold standard diagnostic tool for confirming the echocardiographic findings pertaining to PH (29). Our study showed a significant association of BMI with PH in studies that used RHC for PH diagnosis. Despite being the method of choice for the assessment of PH, the diagnostic use of RHC is limited given its aggressive nature (38).

Several mechanisms can be considered when assessing the association of high BMI with PH. Firstly, hypoxemia, hypercapnia, acidosis, wide swings of intrathoracic pressure, elevated sympathetic tone, and diminished endothelial function are conditions in obstructive sleep apnea and obesity hypoventilation syndrome that lead to pulmonary artery vasoconstriction and subsequent endothelial dysfunction (10). Secondly, excess volume load in the left ventricle due to an increase in the need for metabolically active fat in severely obese patients leads to eccentric left ventricular hypertrophy (38). Over time, impairment of ventricular diastolic filling and changes in ventricular contractility result in increased left atrial filling pressures, which are shifted to the pulmonary venous system (39, 40). Fourthly, insulin resistance-related obesity is linked to deep venous thrombosis and pulmonary embolism (41). Fifthly, diastolic dysfunction or diastolic heart failure secondary to severe obesity generally leads to elevated left ventricular filling pressures with left heart failure, which may increase pulmonary arteriolar remodeling, pulmonary venous pressure, and pulmonary vascular resistance over time (10). Finally, obesity is associated with hyperuricemia, which leads to endothelial dysfunction (10, 42).

This study has some strengths. Based on our knowledge, this study is the first systematic review and meta-analysis evaluating the association of BMI with the risk of PH in adults. Moreover, we did not identify any evidence of publication bias.

This study has several limitations. Firstly, the main limitation is a small sample size. Secondly, although all included studies controlled different types of relevant confounders, it may be necessary to consider other residual confounding factors. Furthermore, because most studies failed to report the confounding factors, we could not perform subgroup analysis according to adjusted factors. Thirdly, the heterogeneity among the studies was extreme, though we tried to minimize it via subgroup analysis. Fourth, the number of studies that provided adequate data for non-linear dose-response analysis was so low that we could not perform this analysis. Fifth, since different form of PH are more likely to be affected by BMI or insulin resistance (such as HFpEF-PH, for example), combining all forms of PH may have error. However, very limited number of articles reported specific form of PH and we could not perform subgroup analysis based on this factor. Finally, combining echocardiogram and RHC derived-definitions of PH is problematic, because, echocardiogram studies used different cut offs, and they do not allow for differentiation of the proper phenotype of PH. However, after excluding studies assessed PH with echocardiogram, small number of studies were remained. Therefore, we included studies assessed PH with either RHC or echocardiogram and performed subgroup analysis for this factor.

In conclusion, we found no significant association between BMI and risk of PH. Further studies are required to confirm these findings.

## Data Availability Statement

The original contributions presented in the study are included in the article/supplementary material, further inquiries can be directed to the corresponding author/s.

## Author Contributions

SP, LY, and JH designed the work. SP and LY extracted the data and wrote the manuscript. BW analyzed the data. JH supervised the work. All authors critically revised and approved the final version of the manuscript.

## Conflict of Interest

The authors declare that the research was conducted in the absence of any commercial or financial relationships that could be construed as a potential conflict of interest.

## Publisher's Note

All claims expressed in this article are solely those of the authors and do not necessarily represent those of their affiliated organizations, or those of the publisher, the editors and the reviewers. Any product that may be evaluated in this article, or claim that may be made by its manufacturer, is not guaranteed or endorsed by the publisher.
